# Characterization of the Cross-Resistance of SARS-CoV‑2 Main Protease Inhibitors, Ibuzatrelvir, Ensitrelvir, and Nirmatrelvir

**DOI:** 10.1021/acsptsci.5c00681

**Published:** 2026-01-28

**Authors:** Haozhou Tan, Xiang Chi, Xufang Deng, Jun Wang

**Affiliations:** † Department of Medicinal Chemistry, Ernest Mario School of Pharmacy, 242612 Rutgers, The State University of New Jersey, Piscataway, New Jersey 08854, United States; ‡ Department of Physiological Sciences, College of Veterinary Medicine, 7618 Oklahoma State University, Stillwater, Oklahoma 74078, United States; § Oklahoma Center for Respiratory and Infectious Diseases, 7618 Oklahoma State University, Stillwater, Oklahoma 74078, United States

**Keywords:** SARS-CoV-2, main protease, ibuzatrelvir, ensitrelvir, nirmatrelvir

## Abstract

The emergence of resistance to SARS-CoV-2 main protease (M^pro^) inhibitors such as nirmatrelvir poses a significant threat to the long-term effectiveness of COVID-19 antivirals. Ibuzatrelvir (PF-07817883) and ensitrelvir are next-generation M^pro^ inhibitors with enhanced metabolic stability, eliminating the need for coadministration with ritonavir, unlike nirmatrelvir. Ibuzatrelvir is currently in Phase 3 clinical trials in the United States, and ensitrelvir is approved in Japan. In this study, we assessed the cross-resistance of ibuzatrelvir, nirmatrelvir, and ensitrelvir against a panel of clinically relevant M^pro^ mutants using FRET-based enzymatic assays, thermal shift binding assays, and cell-based antiviral plaque assays. Our results reveal a cross-resistance pattern of ibuzatrelvir, nirmatrelvir, and ensitrelvir against Q192, S144, H172, and E166 mutants. Notably, the recombinant SARS-CoV-2 virus containing the M^pro^ L50F/E166A/L167F triple mutant is highly resistant to all three drugs in the antiviral plaque assay. These findings underscore the challenge posed by E166 mutations and highlight the need for resistance-resistant M^pro^ inhibitors as future therapeutics.

## Graphical abstract

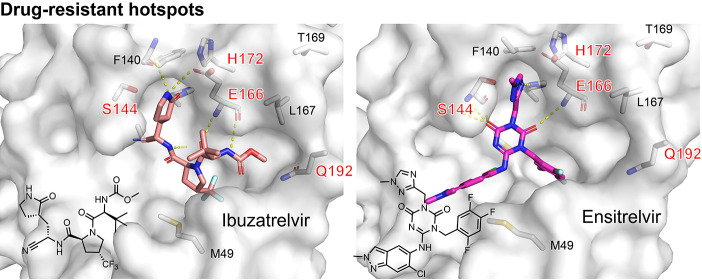

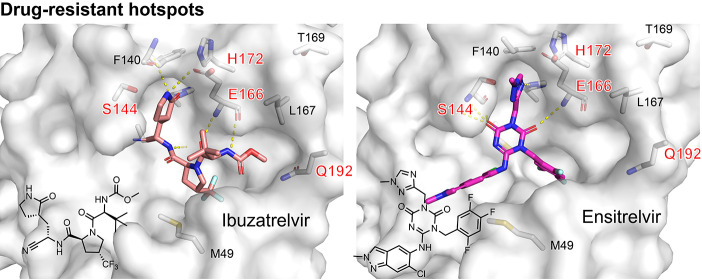

The COVID-19 pandemic profoundly disrupted global health systems, economies, and daily life, leading to millions of deaths and widespread economic and public health impacts. Although the COVID-19 pandemic is subsiding, the virus continues to circulate among humans and animals.^1^ Its ongoing presence poses a persistent public health concern, with potential for the emergence of new variants and future outbreaks.^2^ This ongoing threat underscores the need for additional countermeasures.^3–5^

The SARS-CoV-2 main protease (M^pro^), also known as 3CL^pro^, is an essential viral enzyme that cleaves the viral polyproteins into functional units required for viral RNA replication and translation.^6,7^ M^pro^ plays a critical role in the viral life cycle and has no close human homologues, minimizing the risk of off-target effects.^8^ Due to its sequence conservation and essential function, M^pro^ is a validated and attractive target for the development of antiviral drugs.^6,9^ Inhibiting M^pro^ effectively blocks viral replication, making it a key focus for COVID-19 therapeutics. Intensive academic research and industrial efforts are underway to discover and develop therapeutics targeting the SARS-CoV-2 M^pro^.^4,10^

Nirmatrelvir is an orally active antiviral drug that inhibits the M^pro^.^9,11^ Nirmatrelvir is coadministered with ritonavir as Paxlovid to extend its half-life. It has demonstrated potent efficacy in reducing hospitalization and death in high-risk COVID-19 patients.^12^ Paxlovid received full FDA approval in 2023.^13^ Ensitrelvir (Xocova) is another oral M^pro^ inhibitor drug developed by Shionogi & Co.^14^ It is a nonpeptidic, noncovalent small molecule inhibitor that does not require a pharmacokinetic booster ritonavir.^14^ It has demonstrated efficacy in reducing symptom duration and viral load in patients with mild to moderate COVID-19.^15^ Its limitations include modest efficacy in late-stage trials and transient changes in lipid profiles.^15^ In addition, ensitrelvir is an inhibitor of the cytochrome P450 CYP3A, raising the concern of drug–drug interactions.^16^

Ibuzatrelvir (PF-07817883) is a second-generation M^pro^ inhibitor developed by Pfizer.^17^ It is a peptidomimetic compound derived from nirmatrelvir. Unlike nirmatrelvir, ibuzatrelvir has been chemically optimized for improved metabolic stability, allowing it to be administered without the need for ritonavir boosting.^17^ As of September 2025, Ibuzatrelvir is undergoing Phase 3 clinical trials to evaluate its safety and efficacy in nonhospitalized adults and adolescents with COVID-19 who are at high risk of progressing to severe illness.^18^

Although several candidates have been approved or are in clinical trials, the emergence of drug resistance is an inevitable challenge over time. Antiviral drug resistance typically arises through mutations in the viral genome that alter the target protein, reducing the drug’s binding affinity but maintaining viral replication.^19^ These mutations can occur spontaneously due to the high replication rate and error-prone nature of viral polymerases. Under selective pressure from antiviral treatment, resistant variants gain a survival advantage and become the dominant form.^20^

Nirmatrelvir resistance has been closely monitored and extensively studied, with several resistance hotspots identified.^21–23^ Key mutations in the S1 pocket, particularly at residues E166, S144, and H172, have been associated with reduced drug sensitivity (Figure 1A).^24–26^ The E166V/A mutations, especially in combination with L50F and/or L167F double and triple mutations, have emerged as the most concerning drug-resistant mutants across multiple studies.^21,22,27,28^ The M^pro^ E166V mutant was also identified in human patients treated with nirmatrelvir.^29–31^ In the S2 and S4 pockets, M165 and Q192 contribute hydrophobic interactions with nirmatrelvir, and mutations at these sites have also been linked to resistance in both enzymatic and antiviral assays.^21,32^ Importantly, resistance mutations that retain comparable enzymatic activity and viral fitness to the wild-type (WT) pose significant clinical concern, as they may become prevalent under continued therapeutic pressure from widespread antiviral use.^8^

**Figure 1:**
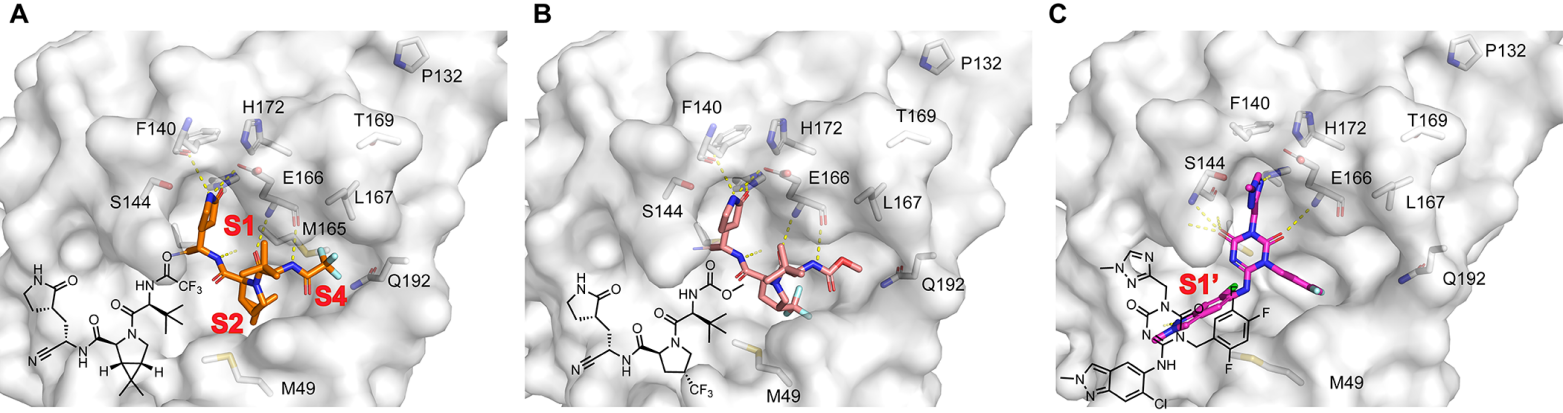
X-ray crystal structures of SARS-CoV-2 M^pro^ with nirmatrelvir (A) (PDB: 7SI9),^33^ ibuzatrelvir (B) (PDB: 8V4U),^17^ and ensitrelvir (C) (PDB: 7VU6).^14^ The figure was created in PyMOL.

Ibuzatrelvir, as a next-generation M^pro^ inhibitor derived from nirmatrelvir, may share cross-resistance due to structural similarities with nirmatrelvir (Figure 1B). Key interactions are conserved between ibuzatrelvir and nirmatrelvir with M^pro^ (Figure 1B).

Ensitrelvir is a noncovalent M^pro^ inhibitor and has a different binding pose from nirmatrelvir and ibuzatrelvir (Figure 1C): the 1-methyl-1H-1,2,4-triazole fits in the S1 pocket and forms a hydrogen bond with the H163 side chain imidazole NH; the 2,4,5-trifluorobenzylic moiety occupies the S2 pocket; while the 6-chloro-2-methyl-2H-indazole fits in the S1′ site.^14^ Both nirmatrelvir and ensitrelvir do not bind to the S1′ site.

Studying the drug resistance profiles of ibuzatrelvir and ensitrelvir is crucial to ensuring sustained efficacy and guiding future therapeutic strategies. In this study, we evaluated the resistance profiles of ibuzatrelvir and ensitrelvir against a panel of well-characterized, nirmatrelvir-resistant SARS-CoV-2 M^pro^ mutants using FRET-based enzymatic assays, thermal shift binding assays, and antiviral plaque assays with recombinant SARS-CoV-2 viruses. Resistance levels were classified based on enzymatic inhibitory constant (*K*_i_) values from the enzymatic assay: moderate resistance (10–100-fold increase) and strong resistance (>100-fold increase). Results revealed a cross-resistance pattern between ibuzatrelvir, nirmatrelvir, and ensitrelvir. Notably, the E166A and E166V associated mutations conferred near-complete resistance to both ibuzatrelvir and nirmatrelvir, highlighting these substitutions as key obstacles in the development of next-generation M^pro^ inhibitors.

## Results

### Omicron Hallmark P132H Remains Sensitive to Ibuzatrelvir, Ensitrelvir, and Nirmatrelvir

The Omicron variant, first identified in 2021, is characterized by markedly increased transmissibility and significant immune evasion, accompanied by a reduction in virulence.^34^ As of 2025, Omicron and its sublineages remain the dominant circulating strains of SARS-CoV-2.^35^ A single amino acid substitution, P132H, has been identified in the predominant M^pro^ mutation of the Omicron variant.^36^ Our previous studies have demonstrated that this mutation does not compromise sensitivity to the protease inhibitor nirmatrelvir.^21,36^ In this study, we characterized cross-resistance of P132H against ibuzatrelvir and ensitrelvir. Enzymatic assays demonstrated that M^pro^ P132H remains sensitive to ibuzatrelvir, with a *K*_i_ of 0.47 nM, which is lower than that of the WT enzyme (Figure 2A and Table S1). Similarly, ensitrelvir displayed consistent inhibition of M^pro^ WT and P132H. Ibuzatrelvir showed dose-dependent stabilization of M^pro^ in the thermal shift assay (TSA) (Figure 2B). In conclusion, the M^pro^ P132H mutant remains sensitive to ibuzatrelvir and ensitrelvir, similar to nirmatrelvir (Figure 2A,B and Table S1).

**Figure 2:**
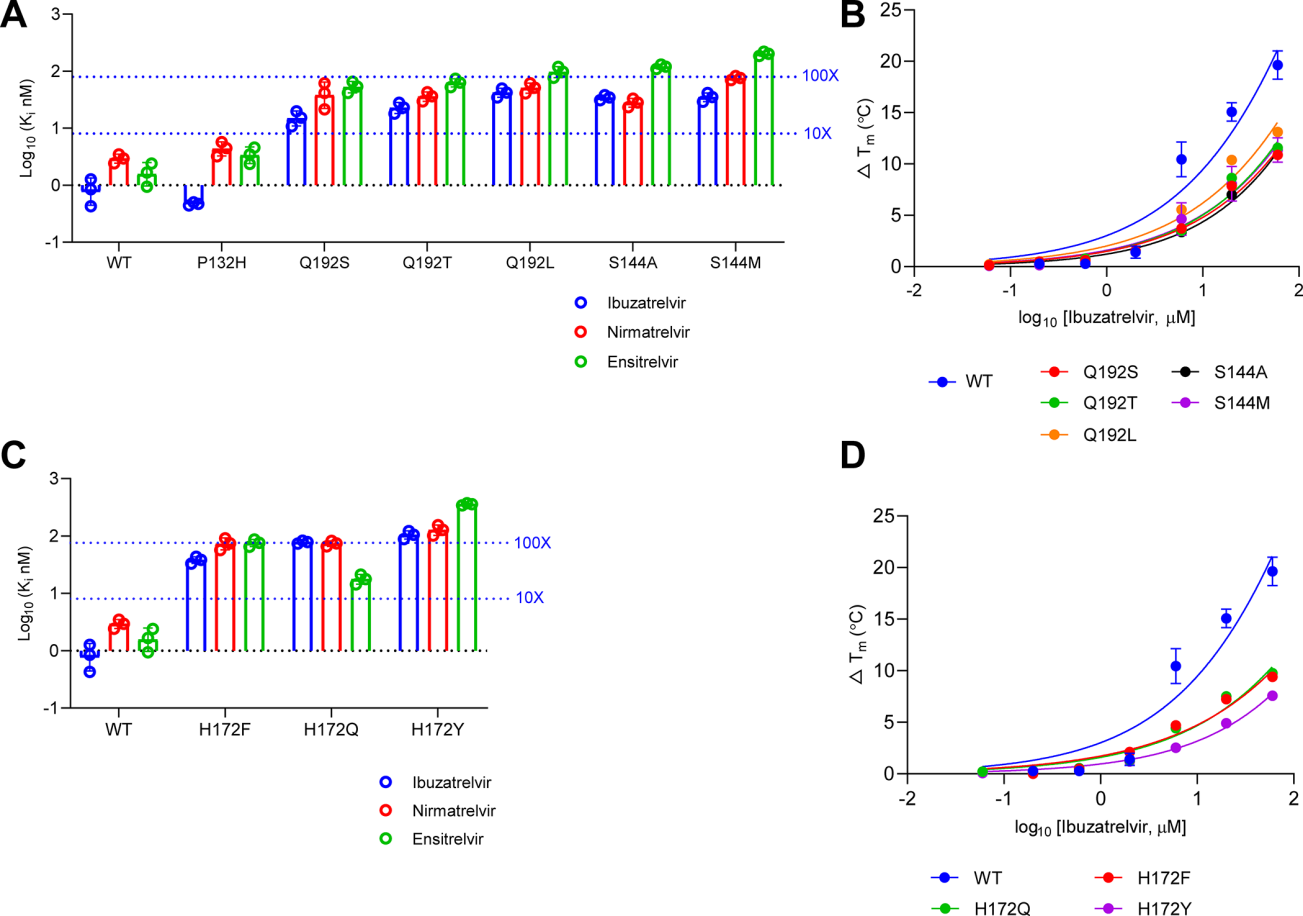
Resistant characterization of SARS-CoV-2 M^pro^ P132H, S144, H172, and Q192 mutants. (A). The inhibitory constant *K*_i_ of M^pro^ P132H, Q192S/T/L, and S144A/M against ibuzatrelvir, nirmatrelvir, and ensitrelvir. (B). The TSA binding assay of M^pro^ Q192S/T/L and S144A/M against ibuzatrelvir. (C). The *K*_i_ of H172F/Q/Y against ibuzatrelvir, nirmatrelvir, and ensitrelvir. (D). The TSA binding assay of M^pro^ H172F/Q/Y against ibuzatrelvir. The *K*_i_ values are the average of three replicates; the error bar represents standard deviation. The reported binding assay results are the change of melting temperature relative to the DMSO-treated group; values are the average of two replicates, and the error bar represents standard deviation.

### Mutations at Residues S144 and Q192 Confer Moderate Resistance to Ibuzatrelvir

Residue S144 is located within the S1 pocket of M^pro^ and does not form a direct interaction with inhibitors (Figure 1). Our previous studies revealed that mutations at this site typically impact drug binding affinity and are often associated with reduced protease fitness.^37^ Among the list of naturally occurring S144 mutants, the S144A and S144M mutants have comparable enzymatic activity to the WT M^pro^, with *k*_cat_/*K*_m_ values within a 10-fold shift.^21^ Notably, both mutations confer 10- to 100-fold resistance to nirmatrelvir.^21^ Given the clinical relevance of these mutations,^38^ we evaluated the inhibitory activity of ibuzatrelvir and ensitrelvir against S144A and S144M. For ibuzatrelvir, the *K*_i_ values were 34.4 nM (S144A) and 35.2 nM (S144M), corresponding to ∼40-fold increases compared to WT. For nirmatrelvir, S144A and S144M exhibited *K*_i_ values of 28.1 and 75.7 nM, representing 10-fold and 27-fold shifts, respectively. These results indicate the moderate resistance of S144A and S144M against ibuzatrelvir and nirmatrelvir. In contrast, markedly higher resistance was observed against ensitrelvir, with *K*_i_ values of 120 nM (72-fold) for S144A and 202 nM (121-fold) for S144M (Figure 2A and Table S1).

Residue Q192 resides in the S4 pocket and contributes to hydrophobic interactions with nirmatrelvir and ibuzatrelvir (Figure 1A,B).^21^ Our earlier work showed that the Q192S, Q192T, and Q192L mutants retain enzymatic activities comparable to WT, with *k*_cat_/*K*_m_ values within a 10-fold difference.^21^ Enzymatic assays revealed that all three mutants exhibit moderate resistance (<100-fold change in *K*_i_) across the inhibitors tested. Specifically, the *K*_i_ values of ibuzatrelvir against Q192S, Q192T, and Q192L were 15.3 (18-fold), 22.9 (27-fold), and 42.2 nM (50-fold), respectively (Figure 2A and Table S1).

To further confirm these resistance phenotypes, TSA was performed to assess direct binding between the M^pro^ variants and ibuzatrelvir (Figure S1 and Table S2). Inhibitor binding typically induces a dose-dependent stabilization of the protease, measured by increases in the melting temperature (Δ*T*_m_). S144M/A and Q192S/T/L mutants showed 1- to 2-fold lower changes in Δ*T*_m_ relative to WT at corresponding ibuzatrelvir concentrations (Figure 2B and Table S2), corroborating the moderate resistance inferred from the enzymatic inhibitory *K*_i_ values. Together, S144A/M and Q192S/T/L mutants confer moderate resistance to ibuzatrelvir with a *K*_i_ value shift between 10- and 100-fold compared to WT.

### H172 Mutations Confer Moderate to Strong Resistance to Ibuzatrelvir and Ensitrelvir

Residue H172 is located within the S1 pocket of M^pro^ but does not form direct interactions with nirmatrelvir or ibuzatrelvir.^21^ Despite this, previous studies have identified H172 as a hotspot for resistance, with mutations such as H172F, H172Q, and H172Y being clinically relevant.^21,39^ These variants retain enzymatic activity within a 10-fold range of WT M^pro^. In our *K*_i_ measurements for ibuzatrelvir, H172F and H172Q showed moderate resistance, with *K*_i_ values of 38.2 nM (45-fold to WT) and 78.4 nM (93-fold), respectively. Similar resistance patterns were observed for nirmatrelvir and ensitrelvir, with all changes remaining within a 100-fold shift compared to WT, classifying them as moderate resistance variants. In contrast, H172Y displayed only moderate resistance to nirmatrelvir (*K*_i_ = 128 nM; 46-fold to WT) but exhibited strong cross-resistance to ibuzatrelvir and ensitrelvir, with a *K*_i_ of 105 nM (ibuzatrelvir, 124-fold) and 357 nM (ensitrelvir, 215-fold) (Figure 2C and Table S1). This prominent resistance to ibuzatrelvir was further supported by the TSA assay, where H172Y showed an overall lower Δ*T*_m_ compared to H172F and H172Q (Figure 2D), indicating weaker binding affinity and enhanced resistance.

### E166 Mutations Confer Dramatic and Cross-Resistance for Ibuzatrelvir, Nirmatrelvir, and Ensitrelvir

Residue E166, located in the S1 pocket of M^pro^, plays a critical role in inhibitor binding by forming three hydrogen bonds with nirmatrelvir and ibuzatrelvir (Figure 1).^21^ Mutations at this position, particularly E166A and E166V, have been reported to confer resistance and are well-established resistant mutations and have been identified by our group and others.^21,28,40,41^

The E166A single mutant displayed moderate resistance to both ibuzatrelvir (*K*_i_ = 66.2 nM; 78-fold shift vs WT) and nirmatrelvir (*K*_i_ = 78.7 nM), and greater resistance to ensitrelvir (*K*_i_ = 216.5 nM; 130-fold). Resistance was further amplified in the E166A/L167F double mutant (Figure 3A,B, Tables S1 and S2). L167F, which resides in the S4 pocket (Figure 1), enhances resistance when combined with E166A. This double mutant exhibited dramatically elevated *K*_i_ values: 1,354 nM for ibuzatrelvir (1612-fold), and 5714 nM for ensitrelvir (3430-fold) (Figure 3A,B, Tables S1 and S2). The L50F substitution, though distal from the inhibitor-binding site, compacts the S2 pocket and enhances hydrophobic interactions with the viral substrate.^42^ It also stabilizes M^pro^ dimerization, thereby increasing the enzymatic activity. L50F frequently coevolves with drug-resistant M^pro^ mutations in viral passage experiments, serving as a compensatory mutation to restore protease fitness.^22^ When combined with E166A and L167F, the resulting triple mutant (L50F/E166A/L167F) retained high resistance to all three inhibitors, with *K*_i_ values of 551.7 nM (ibuzatrelvir; 651-fold), 325.7 nM (nirmatrelvir; 118-fold), and 3,169 nM (ensitrelvir; 1902-fold) (Figure 3A,C, Tables S1 and S2).

**Figure 3:**
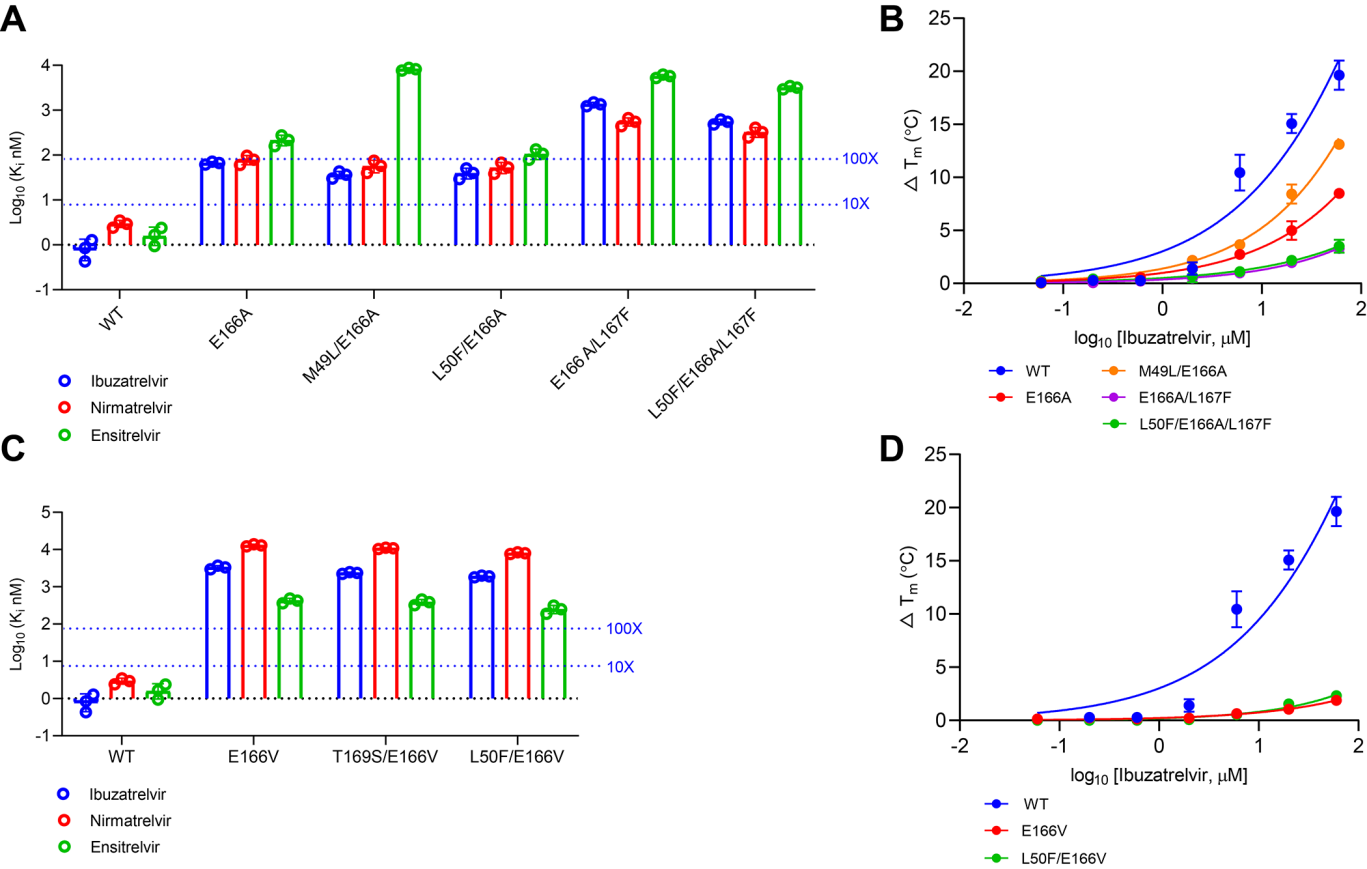
Resistant characterization of SARS-CoV-2 M^pro^ E166 mutants. (A). The inhibitory constant *K*_i_ of M^pro^ E166A and its related double and triple mutants was measured against ibuzatrelvir, nirmatrelvir, and ensitrelvir. Dashed lines indicate the thresholds corresponding to 10- and 100-fold increases in *K*_i_ values. (B). The TSA binding assay of E166A-containing mutants against ibuzatrelvir. (C). The *K*_i_ of E166V and its related double mutants. (D). The TSA binding assay of E166V-containing mutants against ibuzatrelvir. The reported *K*_i_ values are the average of three replicates; the error bars represent the standard deviation. The reported binding assay results are the average of two replicates, and the error bars represent the standard deviation.

E166V is another well-studied resistance mutation, also frequently observed in serial viral passage experiments and human patients.^27,29,31^ Fitness-compensating mutations such as L50F and T169S have been identified in combination with E166V.^43,44^ Our *K*_i_ measurements showed that E166V alone conferred near-complete resistance to ibuzatrelvir (*K*_i_ = 3,319 nM; 3,951-fold). This resistance persisted in double mutants T169S/E166V (*K*_i_ = 2,346 nM; 2793-fold) and L50F/E166V (*K*_i_ = 1895 nM; 2256-fold). Cross-resistance was also observed for nirmatrelvir and ensitrelvir in all E166V-based mutants, each showing over 100-fold increases in *K*_i_ (Figure 3A,C, Tables S1 and S2). Among the three inhibitors, ensitrelvir retained relatively greater potency, both in absolute *K*_i_ values and in fold-shift compared to WT. In terms of the absolute *K*_i_ value, ibuzatrelvir showed better potency against E166V-associated mutants compared to nirmatrelvir (Figure 3C and Table S1).

Lastly, we performed a viral plaque assay to characterize the drug resistance of nirmatrelvir, ibuzatrelvir, and ensitrelvir against recombinant SARS-CoV-2 variants encoding the E166A or E166V mutants.^21^ The plaque assay results showed that rL50F/E166V, rT169S/E166V, and rL50F/E166A/L167F had cross-resistance against ibuzatrelvir, nirmatrelvir, and ensitrelvir (Figure 4A–C). The antiviral resistance profiles are consistent with those observed in the enzymatic and TSA assays (Figure 3). The E166V-containing double mutant viruses, rL50F/E166V and rT169S/E166V, remained partially sensitive to ensitrelvir with EC_50_ value shifts of 7- and 11-fold, respectively, compared to WT. In contrast, for the triple mutant virus rL50F/E166A/L167F, ensitrelvir showed the highest resistance among the three inhibitors, with an 89-fold increase in antiviral EC_50_ value, exceeding the resistance levels of ibuzatrelvir (38-fold) and nirmatrelvir (40.7-fold) (Figure 4C).

**Figure 4:**
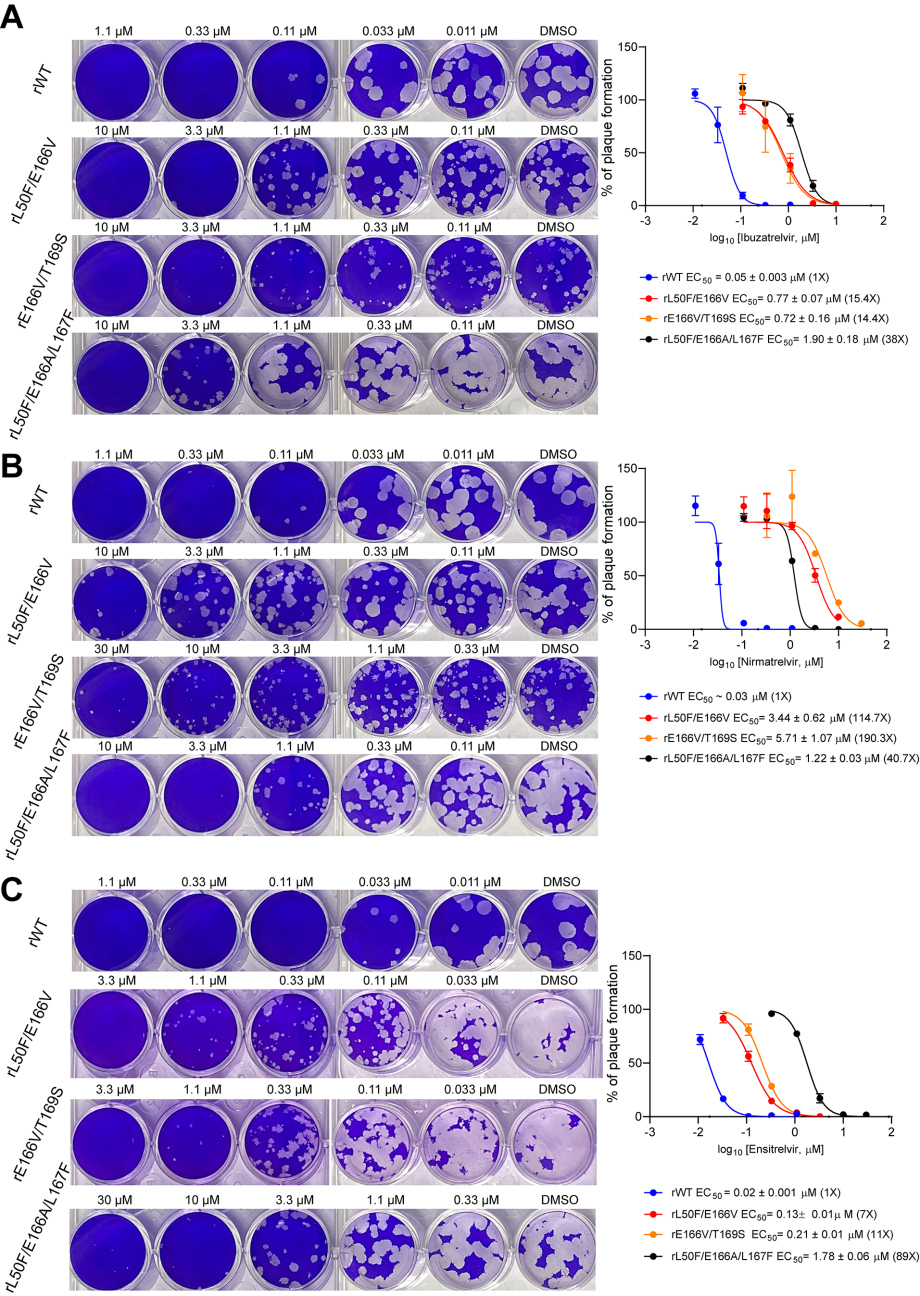
Antiviral plaque assay of nirmatrelvir, ibuzatrelvir, and ensitrelvir against SARS-CoV-2 variants encoding M^pro^ E166 mutations. (A). Plaque assay of recombinant rWT, rL50F/E166V, rE166V/T169S, and rL50F/E166A/L167F against ibuzatrelvir. (B). Plaque assay of recombinant rWT, rL50F/E166V, rE166V/T169S, and rL50F/E166A/L167F against nirmatrelvir. (C). Plaque assay of recombinant rWT, rL50F/E166V, rE166V/T169S, and rL50F/E166A/L167F against ensitrelvir. The images are representative of two replicates. The plotted and reported data are the average of two replicates, and the error bar represents a 95% confidence interval.

## Discussion

The continued evolution of SARS-CoV-2 and the emergence of drug-resistant variants underscore the urgent need for next-generation antivirals with improved resistance profiles.^4^ In this study, we profiled the resistance landscape of ibuzatrelvir and ensitrelvir against a panel of well-characterized nirmatrelvir-resistant M^pro^ mutants using biochemical and cellular antiviral assays. Although many naturally occurring M^pro^ variants have been reported to confer resistance to nirmatrelvir, only a select group, Q192, S144, H172, and E166, maintain WT-like replication fitness and therefore represent the clinically meaningful resistance profile.^21^ Accordingly, our study of cross-resistance focuses specifically on these hotspot residues. All mutants are naturally occurring M^pro^ variants identified in the GISAID database.

Our results reveal that ibuzatrelvir shares a similar resistance pattern with nirmatrelvir and ensitrelvir across major resistance hotspots. Mutations at E166, particularly E166A and E166V, confer the highest levels of resistance to all three inhibitors, confirming E166 as a central vulnerability in current M^pro^-targeting therapeutics. Single E166A and E166V mutations confer strong resistance to ibuzatrelvir (up to ∼4000-fold *K*_i_ shift), and this effect is further amplified when combined with resistant mutations such as L167F in the enzymatic assay. The E166A/L167F double mutant and the L50F/E166A/L167F triple mutant represent some of the most resistant variants identified to date.

Consistent with enzymatic findings, TSAs confirmed the impaired binding of ibuzatrelvir to resistant mutants, as indicated by reduced Δ*T*_m_ values. Furthermore, the viral plaque assay validated that enzymatic resistance translates into reduced antiviral potency in a cellular context. Notably, the plaque assay demonstrated that E166V-based double mutants (L50F/E166V and E166V/T169S) exhibited substantially lower resistance to ensitrelvir compared to ibuzatrelvir and nirmatrelvir. In contrast, the E166A-based triple mutant L50F/E166A/L167F showed high resistance to all three inhibitors, including ibuzatrelvir, nirmatrelvir, and ensitrelvir, highlighting a divergent susceptibility pattern dependent on the specific mutational background. These observations emphasize the importance of using a combination of assays to assess resistance comprehensively.

In addition to E166, we observed moderate resistance associated with mutations at S144, Q192, and H172, most of which maintain a 10- to 100-fold *K*_i_ change to WT. These residues lie in the S1 and S4 binding pockets, modulating inhibitor interactions without significantly compromising protease function. Although the resistance levels conferred by these mutations are not as severe as those of E166 substitutions, their prevalence and preserved catalytic efficiency suggest potential clinical relevance, particularly under sustained antiviral pressure.

Together, our findings highlight the high degree of cross-resistance among structurally related M^pro^ inhibitors and pinpoint E166 as the most critical residue associated with broad-spectrum resistance. These data have several implications for future drug design. First, the development of M^pro^ inhibitors with reduced dependency on E166 for binding may improve resilience against escape mutations. Second, combination therapies or inhibitor cocktails targeting distinct binding sites may help mitigate the emergence of resistance. Finally, continued surveillance and resistance profiling remain essential, as M^pro^ inhibitors become more widely used clinically.

In conclusion, although ibuzatrelvir offers improved pharmacological properties and maintains activity against many nirmatrelvir-resistant variants, it remains vulnerable to key resistance mutations, particularly those at E166. Our study provides a detailed resistance profile that will inform the optimization of future M^pro^ inhibitors and support the strategic management of antiviral resistance in SARS-CoV-2.

## Materials and Methods

### Inhibitors

Nirmatrelvir (HY-138687), ensitrelvir (HY-143216), and ibuzatrelvir (HY-156654) were purchased from MedChemExpress. The purity and molecular weight were confirmed in LC-MS.

### SARS-CoV-2 M^pro^ Mutagenesis, Protein Expression, and Purification

The SARS-CoV-2 main protease (BetaCoV/Wuhan/WIV04/2019) was codon-optimized for *Escherichia coli* expression and cloned into Pet-28a­(+) bearing an N-terminus Hexa-His and SUMO tag. M^pro^ mutant proteins were produced by site-directed mutagenesis using the QuikChange II Site-Directed Mutagenesis Kit (Agilent 200524). The expression vector was transformed into *E. coli* BL21 (DE3) competent cells to overexpress the target protein in Luria–Bertani media at 37 °C. The expression was initiated by the addition of isopropyl β-D-1-thiogalactopyranoside (IPTG) to 0.5 mM with shaking at 18 °C for 16 h. The bacterial culture was harvested by centrifugation and resuspended in lysis buffer (TrisCl 50 mM, NaCl 700 mM, lysozyme 0.5 mg/mL, PMSF 0.5 mM, DNase 0.02 mg/mL, 10% glycerol, pH 8.0). The competent cell was lysed to release the protein by sonication with 35% amplitude on ice with 1 s on and 1 s off for 10 min. The lysate was centrifuged to remove cell debris. The supernatant was incubated with Ni-NTA resin overnight at 4 °C. The Ni-NTA resin was washed thoroughly with 20 mM imidazole in wash buffer (TrisCl 50 mM, NaCl 150 mM, DTT 2 Mm, glycerol 10%, pH 8.0). Then the SUMO M^pro^ was eluted in wash buffer containing 300 mM imidazole. The eluted protein was dialyzed to remove imidazole from the wash buffer. The purified SUMO M^pro^ was digested by SUMO protease I at 30 °C for 1 h. The digested solution was incubated with Ni-NTA resin at 4 °C for 1 h to remove the His-SUMO tag and SUMO protease I. The purified, tag-free Mpro WT and mutants were concentrated using a protein centrifuge filter unit to approximately 10 mg/mL and then fast-frozen in liquid nitrogen, stored at −80 °C.

### Enzymatic Assay

The enzymatic assay was performed using the SARS-CoV-2 M^pro^ FRET substrate Dabcyl-KTSAVLQ/SGFRKME-(Edans). The WT and mutant M^pro^ proteins were diluted to the optimized concentrations in reaction buffer (HEPES pH 6.5, 120 mM NaCl, 0.4 mM EDTA, 4 mM DTT, and 20% glycerol) as previously described.^7,45^ Compounds at various testing concentrations and 20 mM FRET substrate were added to each well of the 96-well plate to initiate the reaction without preincubation. The reaction signal was monitored using a Cytation 5 plate reader (BioTek) using excitation of 360 nm and emission of 460 nm every 70 s for 1.5 h. The first 1 h of initial velocity was obtained by plotting the signal against time with linear regression. The *K*_i_ was obtained by plotting the initial velocity against compound concentrations with the Morrison equation for tight binding in Graphpad Prism. The reported values were the mean of three technical replicates; the error bars represent the 95% confidence interval.

### Thermal Shift Assay

The thermal shift binding assay of SARS-CoV-2 M^pro^ inhibitors was performed using the QuantStudio 5 Real-Time PCR System (Thermo Fisher). M^pro^ WT or mutant proteins were diluted in reaction buffer (HEPES pH 6.5, 120 mM NaCl, 0.4 mM EDTA, 4 mM DTT, and 20% glycerol) to final concentration of 4 μM in 96-well PCR plate and incubated with a series of concentrations of inhibitors at 30 °C for 15 min. 1 × SYPRO orange (Thermo Fisher, Cat no. 4461146) was added to each well, and the signal monitoring was initiated by applying a gradient temperature from 25 to 95 °C with 0.05 °C/s increment. The melting temperature (*T*_m_) was obtained by the Boltzmann model in Protein Thermal Shift Software v1.3. The Δ*T*_m_ was calculated by subtracting the melting temperature of the DMSO-treated group. The reported Δ*T*_m_ values were the average of two technical replicates.

### Cell Line and Viruses

Vero-E6 cells engineered to express human angiotensin-converting enzyme 2 (hACE2) and transmembrane protease serine 2 (hTMPRSS2), designated Vero-AT (NIH-BEI Resources, NR-54970), were maintained in Dulbecco’s modified Eagle’s medium (DMEM) supplemented with 10% fetal bovine serum (FBS), 1% penicillin–streptomycin, 1× nonessential amino acids (NEAA), and 10 μg/mL puromycin (Invivogen, ant-pr-1) to ensure stable expression of the introduced genes. Recombinant SARS-CoV-2 WA1 wild type (rWT) and three previously generated main protease (Mpro) mutant strains (L50F/E166V, E166V/T169S, and L50F/E166A/L167F) were used in subsequent experiments.^3,21^

### Antiviral Plaque Assay

All experiments involving infectious SARS-CoV-2 were conducted within accredited biosafety level 3 (BSL-3) laboratories at Oklahoma State University under protocols approved by the Institutional Biosafety Committee. Personnel performing the work completed comprehensive training in biosafety, biosecurity, and BSL-3 practices and were medically evaluated and cleared through the Oklahoma State University Occupational Health Program prior to engaging in laboratory activities.

Plaque reduction assays were carried out following previously described protocols with minor adjustments.^46,47^ Vero-AT cells were seeded into 12-well CellBIND plates (Corning, 3326) at a density of 1.5 × 10^5^ cells per well approximately 48 h prior to infection. Monolayers were rinsed once with PBS and inoculated with 150 μL of virus suspension containing 40–90 plaque-forming units (PFU) per well. Virus adsorption proceeded for 1 h at 37 °C with gentle rocking every 15 min, after which the inoculum was removed. Each well then received 1 mL of DMEM supplemented with 1.2% Avicel (FMC polymers), a serial dilution of the M^pro^ inhibitor under investigation, and 2 μM CP-100356. CP-100356 is a P-glycoprotein (P-gp) inhibitor and is included to block drug efflux. Because Vero-AT cells express P-gp and many M^pro^ inhibitors are P-gp substrates, adding CP-100356 has become standard practice in SARS-CoV-2 antiviral assays performed in this cell line.^11^ Test compounds (ibuzatrelvir, ensitrelvir, or nirmatrelvir) were dissolved in DMSO and diluted in DMEM using 3-fold serial dilutions. Following a 48–72 h incubation at 37 °C, the overlay medium was discarded, cells were fixed with 4% formaldehyde at room temperature for 30 min, and stained with 0.1% crystal violet for 10 min. Plaques were visualized by photography and quantified using ImageJ software to determine the infected cell areas.

## Supplementary Material



## Notes

Melting temperature of SARS-CoV-2 M^pro^ mutants (Figure S1); the inhibitory constant (*K*_i_) of SARS-CoV-2 M^pro^ mutants (Table S1); Δ*T*_m_ of SARS-CoV-2 Main protease (M^pro^) mutants (Table S2) (PDF)
