# Supplementary material for: Characterization of the Cross-Resistance of SARS-CoV‑2 Main Protease Inhibitors, Ibuzatrelvir, Ensitrelvir, and Nirmatrelvir
Source: ACS Pharmacol Transl Sci. 2026 Jan 28;9(2):404–13. doi: 10.1021/acsptsci.5c00681 (PMC12910490; doi:10.1021/acsptsci.5c00681)
Supplement: Supplementary file 1 [file pt5c00681_si_001.pdf]

Supplementary information

Characterization of the cross-resistance of SARS-CoV-2 main protease inhibitors, ibuzatrelvir, ensitrelvir, and nirmatrelvir

Haozhou Tan,[a] Xiang Chi,[b] Xufang Deng,[b,c,*] and Jun Wang[a,*]

[a]Department of Medicinal Chemistry, Ernest Mario School of Pharmacy, Rutgers, the State University of New Jersey, Piscataway, NJ, 08854, USA

[b]Department of Physiological Sciences, College of Veterinary Medicine, Oklahoma State University, Stillwater, OK, 74078, USA

[c]Oklahoma Center for Respiratory and Infectious Diseases, Oklahoma State University, Stillwater, OK, 74078, USA

*Corresponding authors. Email: junwang@pharmacy.rutgers.edu (J.W.), xufang.deng@okstate.edu (X.D.)

Table of Contents:

Figure S1. **Melting temperature of SARS-CoV-2 Main protease (M^{pro}) mutants**. S2

Table S1. **The inhibitory constant (K_i) of SARS-CoV-2 Main protease (M^{pro}) mutants**. S3

Table S2. **ΔT_m of SARS-CoV-2 Main protease (M^{pro}) mutants**. S4

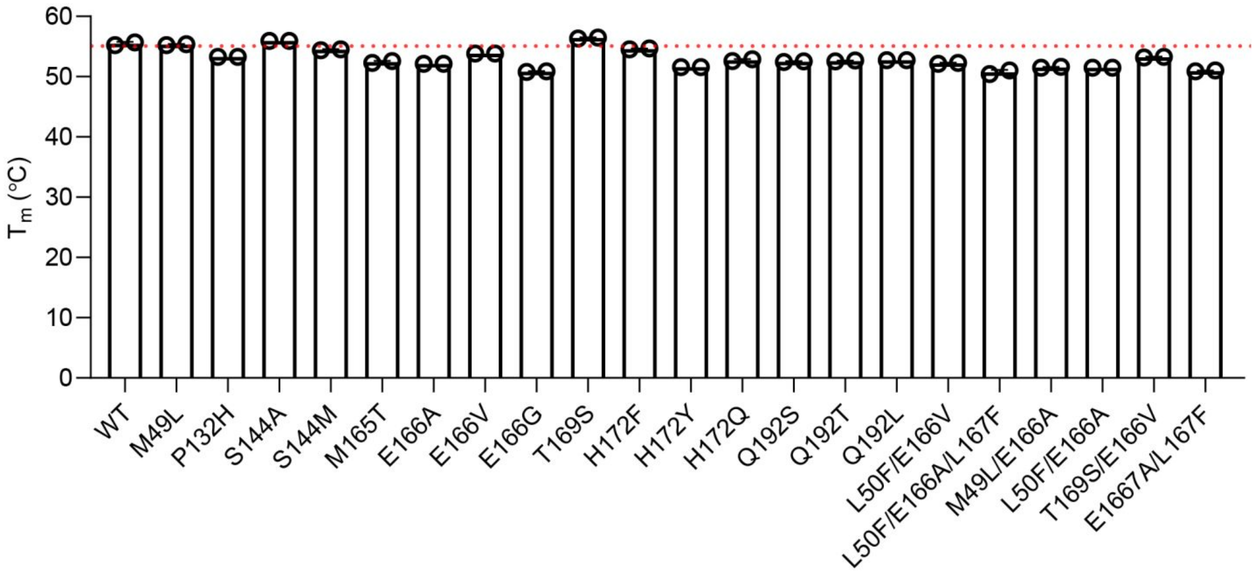

Figure S1. **Melting temperature of SARS-CoV-2 Main protease (M^{pro}) mutants**. The reported results are the average of two replicates.

Table S1. **The inhibitory constant (K_i) of SARS-CoV-2 Main protease (M^{pro}) mutants**. The reported values are the averages of four replicates ± standard deviation.

SARS-CoV-2 M^{pro} Mutants	Ibuzatrelvir K_i (nM)	Nirmatrelvir K_i (nM)	Ensitrelvir K_i (nM)
WT	0.8474 ± 0.42	2.948 ± 0.53	1.666 ± 0.73
P132H	0.471 ± 0.03 (0.5x)	4.446 ± 1.23 (1.5x)	3.46 ± 1.11 (2X)
M49L	0.6514 ± 0.37 (1x)	2.76 ± 0.69 (1x)	102.8 ± 7.73 (62x)
T169S	3.571 ± 0.22 (4x)	7.115 ± 1.54 (3x)	3.876 ± 0.56 (2x)
S144A	34.48 ± 3.57 (41x)	28.1 ± 4.83 (10x)	120.2 ± 10.94 (72x)
S144M	35.22 ± 6.09 (42x)	75.69 ± 6.21 (27x)	202.2 ± 17.24 (121x)
Q192S	15.27 ± 4.50 (18x)	41.31 ± 19.98 (15x)	53.52 ± 12.01 (32x)
Q192T	22.95 ± 4.95 (27x)	36.08 ± 6.64 (13x)	62.94 ± 10.53 (38x)
Q192L	42.19 ± 7.51 (50x)	51.17 ± 10.25 (18x)	97.81 ± 21.10 (59x)
H172F	38.19 ± 5.19 (45x)	74.02 ± 17.22 (27x)	75.74 ± 10.82 (45x)
H172Q	78.42 ± 4.68 (93x)	73.98 ± 8.01 (27x)	17.73 ± 3.45 (11x)
H172Y	105 ± 17.00 (124x)	128.1 ± 26.60 (46x)	357.9 ± 15.69 (215x)
E166A	66.15 ± 4.78 (78x)	78.7 ± 18.20 (29x)	216.5 ± 57.96 (130x)
M49L/E166A	36.44 ± 6.62 (46x)	57.36 ± 17.64 (19x)	8186 ± 586 (4913x)
L50F/E166A	39.76 ± 10.72 (50x)	52.98 ± 14.76 (18x)	106.3 ± 27.85 (66x)
E166A/L167F	1354 ± 133 (1612x)	552.5 ± 111.15 (200x)	5714 ± 507 (3430x)
L50F/E166A/L167F	551.7 ± 71.15 (651x)	325.7 ± 80.46 (118x)	3169 ± 254 (1902)
E166V	3319 ± 358 (3951x)	12950 ± 831 (4404x)	423.1 ± 63.14 (254x)
T169S/E166V	2346 ± 129 (2793x)	10780 ± 472 (3666x)	387.7 ± 67.8 (242x)
L50F/E166V	1895 ± 108 (2256x)	7985 ± 425 (2661x)	250.1 ± 61.7 (156x)

Table S2. **ΔT_m of SARS-CoV-2 Main protease (M^{pro}) mutants**. The reported values are the average of two replicates.

	ΔT_m (°C)					
Ibuzatrelvir (μM)	60 μM	20 μM	6 μM	2 μM	0.6 μM	0.2 μM
WT	20.6	14.4	9.2	0.9	0	0
M49L	19.2	15.4	11.6	1.5	0.4	0.1
S144A	11.2	7.1	3.2	1.4	0.5	0.2
S144M	10.5	6.8	3.5	1.4	0.5	0.1
Q192S	11.1	8.1	3.7	1.4	0.7	0.4
Q192T	11.7	8.9	3.6	1.5	0.6	0.4
Q192L	12.9	10.2	5.1	1.5	0.7	0.4
H172F	9.2	7.0	4.6	2.2	0.4	0
H172Y	7.5	4.7	2.6	1.1	0.4	0.1
H172Q	9.8	7.7	4.6	1.5	0.7	0.4
E166A	8.4	4.3	2.6	1.2	0.7	0.4
M49L/E166A	13.1	9.0	3.7	2.1	0.6	0.0
E166A/L167F	3.2	1.9	0.8	0.4	0.2	0.1
L50F/E166A/L167F	3.9	2.37	1.4	1.1	0.4	0.5
E166V	1.8	0.9	0.7	0.2	0.1	0.1
L50F/E166V	2.3	1.4	0.7	0	0	0
